# History Matters: The Critical Contribution of Historical Analysis to Contemporary Health Policy and Health Care

**DOI:** 10.1007/s10728-017-0348-4

**Published:** 2017-10-23

**Authors:** Sally Sheard

**Affiliations:** University of Liverpool, Liverpool, UK

**Keywords:** Historical analysis, Health policy, Expertise

## Abstract

History is popular with health policymakers, if the regularity with which they invoke historical anecdotes to support policy change is used as an indicator. Yet the ways in which they ‘use’ history vary enormously, as does its impact. This paper explores, from the perspective of a UK academic historian, the development of ‘applied’ history in health policy. It draws on personal experience of different types and levels of engagement with policymakers, and highlights mechanisms through which this dialogue and partnership can be made more efficient, effective, and intellectually rewarding for all involved.

History is popular with health policymakers, if the regularity with which they invoke historical anecdotes to support policy change is used as an indicator. Yet the ways in which they ‘use’ history vary enormously, as does its impact. This paper explores, from the perspective of an academic historian, the development of ‘applied’ history in health policy. It demonstrates, through UK case studies, that historical analysis can improve policymaking and service delivery. By focusing on the actual *process* of policymaking and implementation, especially what happens when earlier policies have been forgotten or deliberately side-lined, historical analysis helps to open up wider opportunities. The paper highlights the similarities between history and improvement science: both disciplines are concerned with change over time, and have developed methodologies to cope with complexity. There is a case to be made for a greater use of historians in health policy and health care, but this requires policymakers and service providers to be aware of what they offer and where to find them, and to routinely engage with them at the start of new policy or service planning. It requires the development of shared objectives, language and planning schedules.

The paper first outlines how history has become a key component of popular culture, especially in the UK, and suggests that this has enabled UK some health policymakers to feel they can ‘do it themselves’ to the exclusion of professional historians. Second, it sets out some of the methodological issues and challenges around historical analysis, especially in the setting of periods of study, in comparison with improvement science. Third, it discusses how historians of healthcare in the UK have chosen their research topics, and crucially, the style of output, and how this has facilitated or hindered their engagement with policymakers and service providers. Fourth, it provides two case studies which demonstrate how history can be used at different scales: at the local level using an anniversary (of the first UK public health team in 1847) to provoke a city (Liverpool) to reflect on what has enabled population health to improve; at the national level (UK Department of Health) to demonstrate the impact of cuts in medical expertise in the civil service (1980s–1990s) on the ability of the government to respond to emerging infectious diseases (HIV/AIDS; BSE; MRSA).

Fifth, it outlines how UK historians are using new methodologies (witness seminars) and new modes of engagement and dissemination (the History and Policy organisation) to become more proactive in working with policymakers. Although history students are now often taught that their discipline is ‘useful’, or has practical significance, because ‘intelligent action’ invariably draws on past experience, some academic historians are unwilling to see historical ‘lessons’ applied to current ‘real-world’ situations.^1^ They would suggest that the uniqueness of a historical event cannot translate perfectly to the present, and so has limited relevance. And, as the US historian Richard Hirsh puts it: ‘More practically, many historians realise that universities rarely provide rewards for work that has direct application outside the ivory tower’.^2^ Yet in the UK historians have been developing external work for many years across policy, creative and other arenas, and indeed ‘impact’ is now a key indicator of success for research councils and for the Research Excellence Framework (REF) that helps determine state funding allocations for universities.

## Challenges of Using History

There are multiple challenges for historians attempting to engage with non-academic audiences, perhaps the first (in terms of importance and sequence) is the issue of superfluity or irrelevance. I have already noted how historians themselves have been reluctant to let their work be used as crude analogy to contemporary circumstances, but equally, some policymakers do not naturally see history as ‘relevant’ to their work, unless provoked to do so. This is due to a number of factors. They will have an understanding of history based on their personal ‘history’—recollections of their school curricula (which in the UK invariably included the stock favourites of Tudors and Stuarts, and the Second World War)—and they will also draw on how they encounter history as entertainment. It has come to dominate TV scheduling in the UK, from mammoth series such as Kenneth Clarke’s *Civilisation* to the new generation of international ‘telly dons’ such as Mary Beard and Simon Schama. History as a genre regularly features in publication bestseller lists (often tied to TV series). This history as entertainment is passive: delivered to the audience, and requiring/allowing little interaction with the historians producing it. Even when historians are invited to deliver serious academic content, their raison d’etre can be misinterpreted. The well-known historian David Starkey gave a keynote speech at the 2006 NHS Confederation conference, but one of the organisers later commented that ‘to some extent, he was there as entertainment’.^3^

The enthusiasm for mainstream history, as evidenced through its presence in the media and bestseller lists also occasionally surfaces within the policymaking community, where policymakers feel confident, perhaps because they have a history degree, that they can identify and apply historical analogies to current policy issues. Sometimes this is well-intentioned, but can also be seen as ‘history as pancea’. Aneurin Bevan, the Labour Minister of Health who oversaw the introduction of the NHS in 1948, has been regularly name-checked by his successors, usually to support politically contentious policy changes, such as hospital closures and service reorganisations.^4^ The repetition of popular historical vignettes, such as the Black Death or the 1918 global influenza pandemic serves to retain and gain them cultural purchase. They also demonstrate preoccupations of their users that are ‘more gothic than historical’.^5^

A second challenge—that of ‘time’—causes concerns for both historians and policymakers. Much of the history that is presented as ‘useful’ or ‘relevant’ by historians when consulted by policymakers (or history self-selected by policymakers themselves) can be labelled ‘contemporary history’. Every period has its own contemporary history, and historians differ on how to set *our* contemporary history’s parameters: whether it covers things within living memory, or as Francis Fukuyama suggests, can be dated from the nineteenth century advent of liberal democracy, in which technology and military competition acted as the twin engines of historical change, as opposed to Marxist theoretical mechanisms of class conflict, politics, and tensions between the individual and the state.^6^ Geoffrey Barraclough provides yet another definition: ‘*Contemporary history begins when the problems which are actually in the world today first take visible shape’* (original italics).^7^ Historians such as John Gaddis and Peter Catterall have championed contemporary history as a way to deepen the historian’s active engagement in the production of history—someone on the ‘inside’—in contrast to E H Carr’s view of the role of the historian as a general watching from the edge of the battlefield as ‘events’ march past him.^8^

Irrespective of how historians conduct their intra-professional debates, for policymakers—especially those politically appointed—the main benefit of using contemporary history context is that it will be familiar to their intended audience: colleagues and/or the public. The supremacy of the recent recognises that ‘every age thinks itself to be the most important age that ever occurred’.^9^

This is more than academic turf wars. It determines how history is ‘done’, both by historians and policymakers, as it delimits what is acceptable evidence, how the analytical frameworks handle it, and its potential for further application. Fernand Braudel (1902–1985), editor of *Annales* and one of the great French historians of the twentieth century, opened a significant debate with his 1958 essay *History and the Social Sciences: the Longue Durée*.^10^ He highlighted the limitations of the historian’s conventional strategy of working with ‘unilinear’ time: in which historical development is presented as continuous on a single (and invariably short) time-scale. This approach, for Braudel, neglected the structures within which change occurred, and prioritised the very recent past. He proposed using an alternative ‘plurality of social time’ in which history operates on three simultaneous levels: the long term (la longue durée: fundamental conditions of life, including the environment); medium term (for social, economic and political systems); the short term (for analysing the individual and the l’histoire événementielle—the ‘event’).

In 1970 an academic symposium on ‘Time’ was convened by the Society for Values in Higher Education that brought together biologists, physicists, psychologists, philosophers and historians. As Dale Porter provocatively stated in his summary of the event, it was the historians who appeared to be least confident in working with the concept of time: ‘In short, historians are working without a viable theory of explanation. Their individual investigations cannot be related to each other in any systematic way, and they are largely irrelevant to studies in other disciplines’. However, their focus on narrative explanations, Dale suggested, were actually similar to the more analytical ‘explanatory models’ used by these other disciplines, and with a bit of effort could be re-purposed to effectively meet the challenges of complex temporal developments, working in a similar way to the ‘process’ philosophy that Alfred North Whitehead had developed earlier in the twentieth century in response to the breakdown in scientific positivism.^11^

A third challenge for using history in policymaking centres on how historical explanation requires relative degrees of abstraction, generalisation and complexity. The main difficulty with narrative accounts is that they involve two kinds of understanding of events. The first is gained by following a sequence of incidents of a given duration, and any pattern abstracted from that narrative is therefore only meaningful by reference to what actually happened. The second kind of understanding—analytical—emerges from a natural tendency to use the pattern abstracted from the story as a heuristic device that prompts questions about the similarity or difference between one sequence of events and another.^12^ These two modes of understanding—by following and by analysing—appeared to be antithetical, and Porter suggested that this duality within traditional historical narrative accounts was at the root of ‘a great deal of anxiety among historians and their critics in recent years’, whereas in other disciplines, especially in the social sciences, these two modes of understanding had been pursued separately. He proposed a conscious re-balancing in which the reciprocity between hypothesis and empirical evidence would validate the historians’ traditional prioritisation of the narrative form of understanding.^13^

Thinking about history as change—of historical events as *dynamic relationships* between causes and effects that happen ‘over time’—raises a fourth challenge which relates more directly to the concept of ‘improvement’, which also has an inherent dynamism. Some weak history—generally that which is uncritical—is branded ‘Whiggish’: it assumes that the present is always better than the past; that there has been progressive, cumulative, ‘improvement’.^14^ In health history/history of medicine, this is often an accurate (if superficial) observation, depending on what measurements are used: life expectancies have improved since the mid-nineteenth century (the upward trajectory only just flattening in the first decade of the twenty-first century). In the fifteenth century more than half the British population were aged under 20; in the early twenty-first century we are already seeing some countries where half the population are over 60. It is tempting to attribute these historical patterns to specific historical events: the introduction of anaesthesia, antisepsis, antibiotics, etc. But when crude national patterns are unpicked it is possible to see that improvement has been relative or unequal: lower mortality rates from the classic infectious diseases such as cholera and smallpox have been paralleled by rising mortality rates for chronic conditions such as diabetes and heart disease. Cancer is in essence a disease of modernity, becoming more visible to society as more human bodies now survive long enough into the new ‘middle ages of 50+’ to allow cell mutations. Underneath Whiggish national improvements in health is an enduring inequality that has been clearly linked to socio-economic factors for nearly two hundred years: Edwin Chadwick, Friedrich Engels and other early nineteenth century reformers were able to demonstrate a significant positive correlation between poverty and ill-health.

Yet ‘long’ health histories—those that begin with the early nineteenth century epidemic disease—are easier, perhaps lazier, and in some ways more politically acceptable, than more recent health history. Politicians are comfortable drawing on the triumph of scientific advances such as anaesthesia and the discovery of germ theory in their rhetoric as evidence of long-term ‘improvement’ in health. To shift to short term history, for the politician, and possibly their policymakers, is more risky. Even using the creation of the NHS in 1948 as a historical era marker raises the potential for political debate on its performance and sustainability. ‘Short’ historical analysis might also be more problematical for historians because it exposes their academic fiefdom to other academic disciplines, especially political scientists and sociologists. It returns us to the first challenge of relevance, and opens up the debate on how to make history useful to policymakers, which requires discussion of historians’ motive, language and agency.

## The Emergence of History of Health and Medicine

The history of health and medicine did not emerge as a distinct sub-discipline before the twentieth century. Rankean historians consolidated their professional identity and status through studies of nation states, in which biographical information on the life (health) and death of key players was a by-product of the bigger narrative.^15^ Studies of the health of populations by professional historians required source material. The introduction of the decadal census in the UK in 1801, and of vital civil registration (births, marriages and deaths) in England and Wales in 1837, enabled a more comprehensive analysis than was possible using the earlier religious archives. Histories of patients, as well as of the great men of medicine, were now possible. There is an irony to this: doctors have always ‘taken histories’ from their patients.

In the US there was an active community of health historians from the 1920s (united through the American Association for the History of Medicine which was formed in 1925, primarily by physicians with an amateur interest in the discipline). US pioneers in the early years, up to the 1960s, included Henry Sigerist, Erwin Ackerknecht and Owsei Temkin. A second generation responded to the concept of a ‘social construction’ of medical authority and the development of medicine as an industrial and commercial activity. Key scholars included Susan Reverby, Charles Rosenberg, Judith Levitt, David Rosner and Rosemary Stevens, to name some of the most prominent proponents. In European countries there were smaller communities of medical/health historians. In Germany it was primarily the province of medical professionals.

Yet a community of historians of health and medicine was not visible in the UK until the 1950s.^16^ The watershed moment of creation of the full British welfare state in 1948 played a critical role in permitting more robust ‘before and after’ historical analysis. Indeed, many of the leading historians working in this area have focused on the achievements of 1948 within their work to support their personal political values. This is not the place in which to digress into a detailed history of the history of health and medicine, but it is useful to outline the leading UK scholars, and a broad split into sub-genres, as this impacts on the UK case studies provided in this paper.

Some, such as Roy Porter and W.F. [Bill] Bynum, focused on science and medicine in the period before the NHS. Others, such as Asa Briggs and Anthony Wohl would be more properly classified as social historians, but their classic books *Victorian People* and *Endangered Lives: Public Health in Victorian Britain* are defining works in the history of public health and early state medicine.^17^ Some historians moved into this area having previously worked on related themes. Charles Webster’s early interests were in early modern science. He then widened his scope to consider medicine in the same period, but it was not until the 1980s that he shifted his focus to the twentieth century, and in particular the health of the working classes.^18^ He was then invited to write the official history of the National Health Service, which appeared in two volumes in 1988 and 1996.^19^

Webster is a ‘proper’ historian, in that he was trained and employed as an academic historian throughout his career. But it is interesting that some of the most influential UK health history books have been written by scholars for whom history was not their ‘day job’. Brian Abel-Smith, a health economist and professor of social administration, produced histories of the British nursing profession and hospitals which remain definitive texts.^20^ These were important political and policy histories of aspects of health care: they were written in support of his socialist principles that a universal free health care system was an intrinsic component of social justice in a civilised society, and a basic human right. Abel-Smith actively used historical context and historical analysis in his work as one of the first special advisers to the British government. He worked closely with Richard Crossman, Barbara Castle and David Ennals, when they served as Secretaries of State for Health during the Labour governments of 1964–70 and 1974–79.^21^ He routinely incorporated history into his briefing papers, and produced anniversary accounts of the NHS to help secure its position at times of increasing financial attacks from the Treasury.^22^

Geoffrey Rivett also used his considerable insider knowledge of the NHS and the DHSS/DH as a former general practitioner and civil servant to write comprehensive histories of the British NHS.^23^ Other health care (service) historians with different backgrounds who were writing in the first phase include Rudolf Klein who initially approached health care from a contemporary public policy perspective, and Nicholas Timmins, who was social policy editor for the Financial Times.^24^ It has also attracted comparative historical analysis through the work of Dan Fox, a US health policy adviser.^25^

## ‘Applied’ History and Historians in the UK

All of the British health care histories noted above, most of them produced in the 1980s and early 1990s, share common features: they are substantial books (most of them more than 300 pages) and their target audiences were primarily the academic community, although some sought wider policymaking and public audiences. Some, such as Abel-Smith, wished to use their work to consolidate and secure the history and future of the NHS; some of the anniversary histories have also been appropriated by politicians and used to legitimise their revisions to Health Minister Aneurin Bevan’s founding principles, while at the same time canonising him within political history (see for example Prime Minister Tony Blair’s foreword to Geoffrey Rivett’s history of the NHS on its 50th anniversary).

Yet it is labour-intensive to take these ‘gold standard’ comprehensive histories of British health care and to use them to support contemporary health policy making. Policymakers prefer short briefing papers and find synthesis of key analysis into bullet points helpful. It is difficult to take even standard academic papers, focusing on specific health care issues, to generate useful ‘policy-applicable’ history. Health care historians, responding to and building on the pioneers listed above, have increasingly chosen to focus on smaller, more manageable themes: hospitals, diseases, clinical innovations, staffing. There has been a trend since the 1990s to push back the period of study to before the creation of the NHS in 1948—to properly examine the ‘mixed economy’ of health care in the inter-war period, which had traditionally been written up as universally bleak.^26^ Another trend has been to look specifically at the history of health care policy development, rather than its delivery and impact.^27^

Alongside this trend for thematic health care histories, has been the emergence of overt ‘policy applied’ health historians. The case studies presented below draw on my personal experiences. Other historians who have increasingly adopted this mantle include Virginia Berridge, whose success is evidenced by her creation and leadership of the first Centre for History in Public Health, at the London School of Hygiene and Tropical Medicine in 2003. Berridge’s career has included significant advisory roles to policymakers working on AIDS, drugs and alcohol policy.^28^ What policy-applied health historians have collectively experienced can be grouped as three key issues: the role of networking to gain access to policymakers; synchronicity with policymaking schedules; style of engagement. These are common issues in the expert/policymaking arena, as evidenced by policy theory literature.^29^

## Case Study 1: Public Health in 1847 and 1997

Liverpool experienced some of the worst epidemics of infectious diseases (cholera, typhus, typhoid) of the early nineteenth century. Its image as a dangerously unhealthy place finally persuaded the urban authority to take action. In 1846, a local Act of Parliament was passed which created the first British public health team, and three men were appointed to start work on 1 January 1847 to deliver a radical programme of sanitary reform (Dr William Henry Duncan as Medical Officer of Health, James Newlands as Borough Engineer, and Thomas Fresh as Inspector of Nuisances). Liverpool continued to develop and implement radical policy solutions through the nineteenth and twentieth centuries, including the first public housing schemes, clean air legislation and attempts at introducing a local ban on smoking in public places. In 1995, John Ashton, then Regional Director of Public Health for North West England, who had previously been a professor of public health at the University of Liverpool, and who had a keen amateur interest in history, commissioned a ‘year of public health’ for 1997 to mark the 150th anniversary of the start of professional public health in the UK.

Ashton supported the creation of a research post in the history of public health at the University of Liverpool, to which I was appointed (later converted into a lectureship). Together we discussed how Liverpool’s health history could be used to generate public debate on the changing determinants of health, especially income levels, unemployment, access to healthcare, and lifestyle factors such as smoking, exercise and diet. This deliberate public engagement policy was intended to be used as leverage with national policymakers on the negative impact of cutting benefits and health services, which had had a disproportionate effect in Liverpool since the election of a Conservative government (under Margaret Thatcher) in 1979. I used the history of local health and healthcare to develop an exhibition with the Museum of Liverpool Life and a programme of ‘celebratory events’, including artistic commissions and activities with local schools. The evaluation at the end of 1997 demonstrated that local awareness of the relative role of health determinants had improved. The city council and local NHS authorities were stimulated by understanding how major policy developments had been achieved by three pioneers in 1847, despite a lack of local funding, staffing or national support. They were encouraged to draw contemporary comparisons and to think more broadly and creatively about solutions to Liverpool’s chronic poor health.

The collaboration between myself and local health policymakers in Liverpool was reported to the UK Department of Public Health. As Liverpool’s pioneering 1846 Sanatory Act was also the foundation for the first national legislation: the 1848 Public Health Act, I was invited to brief the Chief Medical Officer (CMO), Sir Kenneth Calman, and to write a section for his annual report. Working with the US health historian Chris Hamlin, I also presented this history to a wider medical professional audience in an article in the *British Medical Journal* which drew comparisons between the 1848 Act and the plans to re-structure public health in 1998.^30^

## Case study 2: The Decline of the UK Medical Civil Service

Policymakers’ knowledge of where to go for health history is also highlighted through this second case study, which directly leads from the first. When I presented my analysis of the 1848 Public Health Act and its relevance to the 1998 Act at Calman’s final report launch I met the in-coming CMO, Sir Liam Donaldson. He wrote to me afterwards, expressing an interest in the history of the role of the CMO, and inviting me to write a joint research application, which was subsequently funded by the Nuffield Trust.

Between autumn 1998 and the publication of *The Nation’s Doctor: the Role of the Chief Medical Officer 1855*–*1998* in 2006, Donaldson and I met regularly in his Department of Health office in Whitehall, London. We agreed a work plan in which I conducted the historical analysis and wrote the draft chapters, which he then commented on. As the research progressed we discussed emerging issues such as completeness of sources, strengths and weaknesses of oral history, balancing chronological progression with thematic analysis, and historiography: how previous historians had written about CMOs and their work. It emerged that most of the CMOs had faced similar recurrent problems, especially control of adequate staff; relations with the wider civil service and the medical profession; Whitehall culture; the right to speak independently of the government on health issues. These were also issues that Donaldson was then experiencing as the current CMO.

Over the course of 6 years I learnt a lot about how Whitehall works, from reading archive materials deposited by former CMOs, civil servants and politicians, and from discussions with Donaldson about how to achieve policy change, especially on contentious issues such as banning smoking in public places. This was one of his key objectives as CMO, but one which the UK government found tricky as it risked accusations of ‘nanny state-ism’. I researched how previous CMOs had handled the initial discovery of the association between smoking and lung cancer in the 1950s, and then worked towards effective policies to reduce smoking. I demonstrated, through analysis of the archives of the Ministry of Health and the Treasury, that the Conservative governments of Churchill, Eden and Macmillan were reluctant to run effective health education campaigns partly because the relatively new NHS was heavily reliant on revenue from tobacco tax. Sir John Charles, the CMO in post when the association was established, was persuaded not to be too pro-active on this issue. He was allowed to stay in post beyond the usual civil service retirement age because he was ‘amenable’ to pressure from the Treasury, who anticipated that his nominated successor, Sir George Godber, would not be so malleable. Godber did indeed take a much tougher line, and got around the issue of Whitehall policy intransigence by taking it outside government to his medical colleagues in the Royal College of Physicians. He persuaded them to run a hard-hitting, plain English campaign in 1962, which had significant impact in reducing the high levels of smoking in the UK.^31^

The policy lessons from the 1950s history of smoking and lung cancer were indirect but helpful. They illuminated the importance of involving external expertise, and of ensuring senior medical civil servants were not intentionally side-lined by other government departments when financial concerns competed with scientific evidence. These were common ‘history lessons’ that emerged from other research for *The Nation’s Doctor*. One of the case studies that had significant ‘impact’ was on the history of the CMO’s access to medical expertise. From Sir John Simon in 1855 onwards, the CMO had direct line management of medical civil servants, and was able to direct them to conduct rapid investigations into emerging health crises, such as the outbreak of epidemics. This regularly caused friction with the rest of the civil service, especially the Permanent Secretaries in the Ministry/Department of Health, some of whom resented the CMO being able to directly access the Minister without their approval. Sir Arthur Newsholme (CMO between 1908 and 1919) later recorded his frustration that there was:

…an honest belief, common to many government departments, that technical advice is advice not to be given until called for by the secretariat [lay civil service] who, it is assumed, are entirely competent to decide whether such advice is needed. Second, when such advice is on record, it is assumed that it can be safely reapplied in what are regarded by the secretariat as analogous circumstances.^32^

Many of the fourteen men who held the CMO role between 1855 and 1998 found ways to manage this issue, but the last two, Sir Donald Acheson (1984–91) and Sir Kenneth Calman (1991–98) came up against a more substantial obstacle: the successive Whitehall efficiency reviews that from 1979 onwards aimed at shrinking the civil service. These culminated in 1994 in the merger of the previously parallel medical and ‘lay’ civil service reporting hierarchies in the Department of Health, effectively reducing the CMO’s capacity to call upon the support of medical civil servants, at a time of several significant new health threats, including HIV/AIDS, BSE and MRSA. At the peak of the medical civil service in the early 1970s, the CMO had direct line management of over 170 medically qualified civil servants, who provided expertise on the development and implementation of new medical treatments as well as on broader health protection and promotion issues. By the time Calman gave evidence to the BSE enquiry in 1998, he was left with, in his own words, ‘a secretary and a mobile phone’.^33^ I demonstrated, through analysis of archive papers (made available to the Phillips Enquiry on BSE, which would have been otherwise closed under the government’s 30 years rule), and through a series of oral history interviews with senior civil servants, medical professionals and politicians, that there had been a longer decline in medical manpower in Whitehall, and that this had restricted the effectiveness of responses to emerging health crises.

I used the historical evidence I collected on the issue of the shrinking of medical civil service in several ways. First, it formed sections of *The Nation’s Doctor*, co-written with Sir Liam Donaldson. Second, I was approached in 2008 by a medically qualified member of the House of Lords, who had read *The Nation’s Doctor* and wished to use the recent history of the decline of the medical civil service to push the government to invest in staffing and return some of the traditional mechanisms for soliciting external expertise, which had been damaged in the successive Whitehall culls. I re-drafted the analysis for a policy paper published on the website *History and Policy*.^34^ This generated coverage in the *Guardian* newspaper, and a letter from Lord Ara Darzi, then Parliamentary Under-Secretary of State for Health (2007–09). I later learnt from senior civil servants in the Department of Health that the issues I had raised had generated internal debate and an investigation into how to improve institutional memory. Third, I published a longer version of the paper in the academic journal *Social Policy and Administration*, in a more conventional academic format.^35^

## Synchronicity and Styles of Engagement

These personal case studies illuminate the core issues of engagement: the role of networking to gain access to policymakers; synchronicity with policymaking schedules; style of engagement. I have discussed the need to be flexible (accommodating to policymakers’ schedules for meetings, deadlines and focus of outputs). It is worth saying more about the issues of synchronicity and styles of engagement. From my other experiences of working with policymakers I have come to appreciate the need to respond quickly: policymakers work on much shorter timescales, often looking for analysis results within weeks or months. Historians, especially those engaged on large projects, will be more comfortable with planning their research over years rather than months. The conventional format of outputs as monographs (prioritised by the periodic Research Excellence Framework [REF]), and academic journal articles take months or years to write and then work their way through rigorous peer review systems, which then need to be fitted into publishing schedules.

History and Policy, a UK web-based academic organisation, was founded in 2002 partly in response to this ‘constipated’ academic output culture.^36^ It now has a membership of over 500 historians, who are encouraged to work up their research into short Policy Papers and Opinion Pieces. These go through quick peer review for rapid web-based publication. The papers are carefully targeted at policymakers and journalists by the organisation’s press officers. Where possible, publications are scheduled to coincide with anticipated government policy statements. The policy papers begin with executive summaries, in the style of policy briefing papers. They contain essential historical context, and provide suggested further sources. History and Policy has developed from its early focus on presenting academic history in a new format, to deliver bespoke services to government departments (seminar series and workshops). It engages directly with policymakers; responds quickly to invitations to collaborate, for example, providing historical content for the No. 10 Downing Street [Office of the Prime Minister] website; and supports historians in preparing written submissions to parliamentary enquiries.^37^

Other new styles of engagement between historians and policymakers have been developed within the last couple of decades. Witness seminars provide opportunities for people who were involved in significant policy decisions to come together and reflect on the process and outcomes. The Wellcome Trust has been one of the most active pioneers of this format, convening more than 50 witness seminars on a wide range of health policy issues, from development of hip replacements to epigenetics.^38^ Early career historians are offered training in public and policy engagement (History and Policy regularly run workshops, funded by the main research councils), and some are able to take up placements in government departments.

The issue of location of historians is also critical. While most historians are based within traditional university history departments, there is a strong case to be made for ‘embedding’ historians within other academic departments and in external policymaking environments. This provides more opportunities for serendipitous encounters, and encourages policymakers to seek advice. For historians, the benefit of having other homes is that they are there at the moments when policy shifts become apparent, and can respond efficiently. They can develop the necessary language of the policymaking organisation and knowledge of how policy ‘gets done’: the complexities of local and national government committees and reviews, the importance of knowing key staff. These issues are of course not specific to historians, but relevant to all expert policy advisers, and are analysed in Richard Freeman and Steve Sturdy’s excellent edited book, *Knowledge in Policy: Embodied, inscribed, enacted*.^39^

## Conclusion

Despite the considerable progress made by historians in engaging with health policymakers in the UK, it is important to be realistic on the authority of historical knowledge in the policy arena. It competes with analyses of the current situation, and predictions of possible future scenarios. The mode of engagement—usually crisis-driven if initiated from the policy community—de-limits a ‘context-only’ or ‘passive–reactive’ response from the historian. Yet history used to be the default policy science.^40^ Although historians are still often asked for ‘facts’ rather than analysis, they are getting better at initiating a more useful mode of engagement.^41^ Good examples come from the recent health and social care scandals of Mid-Staffordshire Hospital and those triggered by the exposure of Jimmy Savile’s abuse of vulnerable children and adults. Historians from the History and Policy organisation were invited to give evidence to the Savile public inquiry, and several opinion pieces were written that addressed the ‘never again’ issue behind these scandals.^42^ However, we rarely reach a critical tipping point that permits policy change *on the primary basis of* historical evidence.^43^ Historical analysis has not yet become an integral and *initial* part of policy planning process.

Robust and routine historical analysis of new policy issues can add real value.^44^ It permits discussion of some of the bigger, or neglected questions in health and health care, such as choice of funding mechanisms, expectations and responsibilities (by both the public and the state). It can illuminate more fundamental issues, such as health as a human right, and demonstrate the implications of politicising access to health care, that may have evolved over centuries, rather than the years which usually delimit the frame of reference for policymakers. Historical analysis can explain the persistence of institutional structures (the NHS) and cultural attitudes (the British public’s love for the NHS) and expose how policymakers use history to reinforce or disrupt the status quo. Its preference for subjective, intuitive analysis (of texts, oral history) can cope with the finer nuances involved in policymaking: the ‘what if’ and ‘so what’ quandaries. It enables a better understanding of the policy development process, and through specific case studies show what happens when potential policies get forgotten or deliberately side-lined.^45^ Historians are skilled at handling complexity: there are significant similarities between what historians, improvement scientists and policymakers do. What needs to be further developed are the shared objectives and language to enable a more rewarding collaboration.

## Footnotes

1 J. Tosh and S. Lang, *The Pursuit of History: Aims, Methods and New Directions in the Study of Modern History* (New York 4th edition, Longman Pearson, 2006), p. 1–2.

2 R. Hirsh, ‘Historians of Technology in the Real World: Reflections on the Pursuit of Policy-Oriented History’, *Technology and Culture* 52; 1 (2011); 6–20.

3 V. Berridge, ‘History Matters? History’s role in health policymaking’, *Medical History* 52 (2008); 311–326.

4 See for example how the historian Charles Webster took the Labour government to task in 2002 for rewriting NHS history to suit its current reform plans: C. Webster, ‘The Parable of the Incompetent Steward’, *British Journal of Health Care Management* 8; 3 (2002); 113–14.

5 A. Bashford and C. Strange, ‘Thinking historically about public health’, *Medical Humanities* 33 (2007): 87–92. ‘Gothic’ is used here to mean a preoccupation with events that are portentously gloomy or horrifying.

6 F. Fukuyama, *The End of History and the Last Man* (Harmondsworth, Penguin, 1992); P. Catterall, ‘What (if anything) is Distinctive about Contemporary History?’, *Journal of Contemporary History* 32; 4 (1997); 451.

7 G. Barraclough, *An Introduction to Contemporary History* (Harmondsworth, Penguin, 1967), p. 20.

8 J.L. Gaddis*, On contemporary history. An inaugural lecture delivered before the University of Oxford 18 May 1993* (Oxford, Clarendon Press, 1995); P. Catterall, ‘What (if anything) is Distinctive about Contemporary History?’, *Journal of Contemporary History* 32; 4 (1997); 444–52.

9 J.L. Gaddis*, On contemporary history. An inaugural lecture delivered before the University of Oxford 18 May 1993* (Oxford, Clarendon Press, 1995), p. 21.

10 F. Braudel, ‘History and the Social Sciences: *la longue durée’*, 1958, reprinted in F. Braudel, *On History* (trans. Sarah Matthews) (Chicago, University of Chicago Press, 1980). See also J. Tosh (with S Lang), *The Pursuit of History* (Harlow, Pearson Education Ltd, 1984).

11 D. Porter, *The Emergence of the Past. A Theory of Historical Explanation* (Chicago, University of Chicago Press, 1981), p. ix.

12 Porter, *Emergence of the Past*, pp. 29–31.

13 Porter, *Emergence of the Past*, p. 2.

14 The term comes from the Whigs—a British political party active from the seventeenth to the nineteenth century.

15 The German historian Leopold von Ranke (1795–1886) was influential in setting standards for historical methodologies, especially the use of primary sources and an emphasis on narrative history and international politics.

16 As one reviewer pointed out, this was also the period when medical ethics, bioethics and medical law emerged as distinct disciplines. See D. Wilson, ‘What can History Do for Bioethics?’, *Bioethics* 27;4 (2013); 215–223; J. Harrington, Towards a Rhetoric of Medical Law (London, Routledge, 2016).

17 A. Briggs, *Victorian People* (1983); A.S. Wohl, *Endangered Lives: Public Health in Victorian Britain (London, Dent, 1983).*

18 C. Webster, *The Great Instauration: Science, Medicine and Reform 1626–1660* (New York, Holmes & Meier, 1975); C. Webster, *Health, Medicine and Mortality in the Sixteenth Century* (Cambridge, Cambridge University Press, 1979); C. Webster, *Biology. Medicine and Society 1840-1940* (Cambridge, Cambridge University Press, 1981); C. Webster, ‘, ‘Healthy or Hungry Thirties?’, *History Workshop Journal*, 13, 1 (1982); 110–29.

19 C. Webster, Problems of Health Care: the National Health Service Before 1957 (London, HMSO, 1988); C. Webster, Government and Health Care: the National Health Service 1958–1979 (London, The Stationery Office, 1996).

20 B. Abel-Smith, *A History of the Nursing Profession* (London, Heinemann, 1960); B. Abel-Smith, *The Hospitals 1800-1948* (London, Heinemann, 1964).

21 S. Sheard, *The Passionate Economist. How Brian Abel-Smith shaped global health and social welfare* (Bristol, Policy Press, 2013).

22 A. Abel-Smith, *The National Health Service: the First Thirty Years* (London, HMSO, 1978).

23 G. Rivett, *The Development of London’s Hospital Systems* (1986); G. Rivett, *From Cradle to Grave: 50* *Years of the NHS* (London, King’s Fund, 1998).

24 R. Klein, *The Politics of the National Health Service* (London, Longmans, 1983); N. Timmins, *The Five Giants: A Biography of the Welfare State* (London, Fontana, 1996).

25 D. Fox, *Health Policies, Health Politics: the British and American Experience, 1911–1965* (Princeton N.J., Princeton University Press, 1986).

26 S. Szreter, ‘Health, Class, Place and Politics; social capital and collective provision in Britain’, *Contemporary British History* 3(2002); 27–57; J. Stewart, ‘Ideology and Process in the Creation of the British National Health Service’, *Journal of Policy History* 14 No. 2 (2002); 114–134; M. Gorsky and S. Sheard (eds), *Financing Medicine: The British Experience since 1750* (Routledge, 2006); A. Levene, M. Powell, J. Stewart and B. Taylor, *Cradle to Grave: Municipal medicine in Inter-war England and Wales* (Peter Lang, 2011); M. Gorsky, ‘Local Government Health Services in Interwar England: Problems of Quantification and Interpretation’, *Bulletin of the History of Medicine* 2011 (85), 384–412; M Gorsky, ‘The British NHS 1948–2008: A review of the historiography’, *Social History of Medicine* 21 (2008), 437–60.

27 V. Berridge (ed.), *Making Health Policy: Networks in Research and Policy after 1945* (Amsterdam, Rodopi, 2005).

28 Berridge, ‘History Matters?’ (2008); V. Berridge and J. Stewart, History: a social science neglected by other social sciences (and why it should not be), *Contemporary Social Science* 7; 1 (2012); 39–54.

29 E. Perdiguero, J Bernabeu, R. Hertas and E. Rodriguez-Ocana, ‘History of health, a valuable tool in public health’, *J. Epidemiology and Community Health* 55 (2001); 667–673; S. Sheard, ‘History in health and health services: exploring the possibilities’, *J. Epidemiology and Community Health* 62 (2008); 740–744; R. Freeman and S. Sturdy (eds), *Knowledge in Policy: Embodied, inscribed, enacted* (Bristol, Policy Press, 2015).

30 C. Hamlin and S. Sheard, ‘Revolutions in public health: 1848, and 1998?’, *British Medical Journal* 317 (1998); 587–91.

31 Royal College of Physicians of London, *Smoking and Health: report of the Royal College of Physicians of London on smoking in relation to cancer of the lung and other diseases* (London, Pitman, 1962).

32 A. Newsholme, *The Last 30* *Years in Public Health* (London, George Allen and Unwin, 1936), p. 62.

33 British Medical Journal editorial, ‘Staff cuts would leave the CMO stranded’*, British Medical Journal* 317 (1998); 232.

34 S. Sheard, ‘Doctors in Whitehall: how the government manages medical advice at the 60^th^ anniversary of the NHS, www.historyandpolicy.org.uk (last accessed 27.12.16).

35 S. Sheard, ‘Quacks and Clerks: Historical and Contemporary Perspectives on the Structure and Function of the British Medical Civil Service’*, Social Policy and Administration* 44;2 (2010), 193–207.

36
http://www.historyandpolicy.org/. Last accessed 2.2.17.

37 M. Gorsky, Memorandum submitted to the Health Select Committee Inquiry into Public and Patient Involvement in the NHS.http://www.historyandpolicy.org/hp/docs/gorsky_memo.pdf/. Last accessed 2.2.17.

38
https://blog.wellcome.ac.uk/2014/09/24/21-years-wellcome-witness/. Last accessed 2.2.17.

39 Freeman and Sturdy, *Knowledge in Policy*.

40 V. Berridge and J. Stewart, ‘History: a social science neglected by other social sciences (and why it should not be)’, *Contemporary Social Science* 7; 1 (2012); 39–54; E. Fee and D. Fox, ‘Introduction: AIDS, public policy and historical enquiry’, in E. Fee and D. Fox (eds*), AIDS and the burdens of history* (Berkeley, CA, University of California Press, 1988), 1–11.

41 V. Berridge, Public or Policy Understanding of History? *Social History of Medicine* 16; 3 (2003); 511–523.

42 See also the US historian Allan Brandt’s contributions as an expert witness in a landmark 2006 court case involving tobacco companies, in which he explained how they manipulated the scientific debate over the risks of smoking in the 1950s. A.M. Brandt, ‘From analysis to advocacy: crossing boundaries as a historian of health policy’, in F. Huisman and J.H. Warner (eds), *Locating Medical History* (Baltimore, Johns Hopkins University Press, 2004). A. Brandt, *The Cigarette Century: The Rise, Fall and Deadly Persistence of the Product that Defined America* (New York, Basic Books, 2009).

43 C. Hilton, ‘Whistle-blowing in the National Health Service since the 1960s’, History and Policy paper published 26.8.16. http://www.historyandpolicy.org/policy-papers/papers/whistle-blowing-in-the-national-health-service-since-the-1960s. Last accessed 2.2.17; S. Sheard, Can we never learn? Abuse, complaints and inquiries in the NHS’, History and Policy paper published 26.2.15. http://www.historyandpolicy.org/opinion-articles/articles/why-we-never-learn-abuse-complaints-and-inquiries-in-the-nhs. Last accessed 2.2.17; A. Bingham, L.Delap, L. Jackson and L. Settle, ‘” These outrages are going on more than people know”’, History and Policy paper published 26.2.15. http://www.historyandpolicy.org/opinion-articles/articles/these-outrages-are-going-on-more-than-people-know. Last accessed 2.2.17.

44 For a US perspective see J. Zelizer, Clio’s Lost Tribe: Public Policy History since 1978’, *Journal of Policy History* 12 (2000); 369–94.

45 R.E. Neustadt and E.R. May, *Thinking in Time: the Uses of History for Decision Makers* (New York, Free Press, 1986).

